# Retinal neurovascular alteration in type 2 diabetes with renal impairment in association with systemic arterial stiffness

**DOI:** 10.1186/s40942-023-00521-5

**Published:** 2024-01-02

**Authors:** Sauli Ari Widjaja, William F. Mieler, Wimbo Sasono, Soebagijo A. Soelistijo, Arief S. Kartasasmita, Akira Murakami, Shintaro Nakao

**Affiliations:** 1 Department of Ophthalmology, Faculty of Medicine, Universitas Airlangga/ Dr. Soetomo General Academic Hospital, Jl. Mayjen. Prof. Dr. Moestopo 6-8, Gubeng, 60286 Surabaya, East Java Indonesia; 2 Department of Ophthalmology, Juntendo University Graduate School of Medicine, Tokyo, Japan; 3 Department of Ophthalmology and Visual Sciences, University of Illinois at Chicago, Chicago, USA; 4 Department of Internal Medicine, Faculty of Medicine, Universitas Airlangga/ Dr. Soetomo General Academic Hospital, Surabaya, East Java Indonesia; 5 Department of Ophthalmology, Faculty of Medicine, Universitas Padjadjaran/ Cicendo National Eye Hospital, Bandung, West Java Indonesia

**Keywords:** Retinal neurovascular, Arterial stiffness, Renal impairment, Diabetic retinopathy, baPWV, OCT/OCTA, Pulse wave velocity, Diabetes, Cardiovascular disease

## Abstract

**Background:**

Diabetic retinopathy (DR) patients should be alert for subclinical macroangiopathy. We aimed to investigate the association between retinal neurovascular alteration and systemic arterial stiffness in type 2 diabetes mellitus (type 2 DM) patients with varying degrees of renal impairment.

**Methods:**

The study included 170 patients with confirmed diagnosis of type 2 DM aged ≥18 years old. Renal function was assessed by estimated glomerular filtration rate (eGFR). Arterial stiffness was measured by brachial-ankle pulse wave velocity (baPWV) and ankle brachial index (ABI). Retinal neurovascular parameters were derived from Optical Coherence Tomography (OCT)/OCT-Angiography, represented by vessel density (VD Central, Inner, Outer, Full), foveal avascular zone (FAZ area and FAZ perimeter) of the superficial capillary plexus, the average of macular ganglion cell-inner plexiform layer thickness (ave mGC-IPLt) and the average of retinal nerve fiber layer thickness (aveRNFLt). The association between variables among the groups (according to renal function, diabetic retinopathy (DR) severity, and arterial stiffness categories) were analyzed by regression analysis with multiple hypothesis testing commands.

**Results:**

Out of the 265 eyes, the mean DM duration and HbA1c were 6.21 ± 6.37 years and 8.44 ± 2.06% respectively. While the mean of eGFR, baPWV and ABI were 66.78 ± 32.80 ml/min/1.73m2, 15.49 ± 3.07 m/s, and 1.05 ± 0.12, respectively. Patients with more severe renal impairment demonstrated longer DM duration (*p* < 0.001), higher baPWV (*p* < 0.0001), and retinal vascular alteration. Proliverative DR group showed the lowest eGFR (*p* < 0.0001), highest baPWV (*p* < 0.0001), and retinal neurovascular changes. Significantly lower eGFR and retinal vascular alteration were found in the baPWV > 14 group. Some neurovascular parameters were significantly negatively correlated with baPWV; moreover, retinal neurovascular changes were also noted in the abnormal ABI group.

**Conclusions:**

The strong association between changes in the retinal neurovascular system, DR severity, renal impairment, and arterial stiffness in type 2 DM was confirmed. Patients with more severe renal impairment had higher levels of arterial stiffness, more severe DR and retinal neurovascular alteration. Retinal neurovascular changes seen in OCT/OCTA might mimic renal microvascular alteration and systemic arterial stiffness. Therefore, assessment of baPWV and OCT/OCTA should be integrated in DR screening to enhance cardiovascular risk stratification and prognosis as well as to provide clinically useful early identification of subclinical micro- and macrovascular alterations.

## Backgrounds

Indonesia is expected to have more than 20 million people with diabetes mellitus (DM) by 2030 [1]. From the Jogjakarta Eye Diabetic Study in the Community (JOGED.COM) in Indonesia, it is found that diabetic retinopathy (DR) affects 43.1% of the population, with 26.3% vision threatening DR (VTDR) responsible for 7.7% bilateral blindness [2]. As the prevalence of diabetes rises, the prevalence of diabetic microvascular complications, such as DR and diabetic kidney disease (DKD), is predicted to increase and the risk factor of cardiovascular disease (CVD) mortality is higher in DKD and DR [3, 4]. Therefore, more specific approaches for early identification of people at risk of DR, DKD, and CVD, especially in non-invasive assessments, are urgently needed [4]. In this respect, the predictive relevance of arterial stiffness on systemic CVD and its use as the primary tool to screen metabolic syndrome has been suggested by a number of research works undertaken in recent decades [5–7]. Non-invasive measurements of the brachial-ankle pulse wave velocity (baPWV) and ankle-brachial index (ABI) have been used to quantify arterial stiffness [8].

The similarities between the eye and the kidney allow non-invasive retinal neurovascular imaging techniques, such as optical coherence tomography (OCT) and OCT-angiography (OCTA) assessment, to detect microvascular abnormality to better identify at-risk individuals [9]. Some recent studies focused on the association between renal function and retinal-neural or microvascular changes derived from OCT/OCTA in type 2 DM [10–12]. While another study from Xueyang Zhang et al. demonstrated arterial stiffness increase in the group with early diabetic nephropathy [13]. Further, some previous studies focused on the association between arterial stiffness and the presence or the severity of DR [14, 15] using different methods of measurement, such as carotid-femoral pulse wave velocity (cfPWV) [16] or ABI [14], and one study exhibits correlation between arterial stiffness using cardio-ankle vascular index (CAVI) with chorioretina microvasculature [17]. However, the significance of arterial stiffness for early detection in combination with retinal neurovascular assessment of diabetes with renal impairment still need to be explored.

The concept of DR as a microvascular disease has evolved with more emphasis on the involvement of the neurovascular unit, including microvascular abnormalities and neural component degeneration that plays significant role [18, 19]. The underlying mechanism and association between arterial stiffness and diabetic ocular neurovascular alterations remain unknown. In this study, we would like to investigate the association between retinal neurovascular alterations and systemic arterial stiffness as measured by baPWV and ABI in type 2 DM patients with varying degrees of renal impairment. Despite encouraging results being reported, to the best of our knowledge, very limited studies linking and analyzing the retinal neurovascular parameter, with a broad array of systemic conditions, include arterial stiffness and renal impairment in type 2 DM patients. Moreover, expanding the analysis collectively would further provide a better depiction of the at-risk patient of DR, DKD, and CVD, since they are not only at risk of losing their sight but also at higher risk of developing life-threatening systemic vascular and renal complication of diabetes.

## Methods

### Study design

This cross-sectional single-center study was conducted by Diabetic Ocular Renal Surabaya Study (DiORS Study) group at The Endocrinology and Diabetes Outpatient Department (ED-OPD) and Ophthalmology Outpatient Department, Dr. Soetomo General Academic Hospital (DSGAH) Surabaya, Indonesia from August 2019 to January 2020. This study was supported by the Universitas Airlangga Research Grant (Grant SK Rektor Unair No.1408/UN3/2019). The study protocol was approved by the institutional review board of the DSGAH (Komisi Etik Peneliti Kesehatan Rumah Sakit Dr. Soetomo No.1311/KEPK/VII/2019). All clinical investigations were carried out in accordance with the principles outlined in the Declaration of Helsinki, and written informed consents were obtained from all patients.

### Study population

Patients with confirmed diagnosis of type 2 DM aged ≥18 years old who visited ED-OPD during the study period and who were willing to sign the informed consent were included in the study. Only 170 of the 201 patients who provided informed consent underwent all examinations. The excluded patients had a history of malignancy, hemodialysis treatment or renal transplantation, were an active smoker, used dyslipidemia medication, and had incomplete medical records. After a thorough examination in ED-OPD, arterial stiffness measurement and laboratory tests were performed, the patients then were referred to ophthalmology OPD for ocular assessment. Ocular exclusion criteria included the history of eye surgery, high myopia, glaucoma suspect, history of laser treatment, history of intravitreal injection and poor-quality image. Ocular examination, laboratory tests, and arterial stiffness measurement were performed within a 1-month time frame.

### Collection of demographic, clinical characteristic, and laboratory data

Demographic and clinical characteristics, including age, gender, smoking status, history of medication use, DM duration, hypertension (HT), systolic blood pressure (SBP), diastolic blood pressure (DBP), heart rate (HR), body mass index (BMI), hemoglobin A1c (HbA1c ) levels, lipid profile (total cholesterol, high-density lipoprotein (HDL), low-density lipoprotein (LDL), and triglycerides (TG)), renal function (creatinine serum (Cr) and the estimated glomerular filtration rate (e-GFR) that was calculated by the Modification of Diet in Renal Disease (MDRD) formula)) (Levey AS et al.,1999), were obtained. According to eGFR level, in this study, cases were divided into three groups: CKD st.1–2 (eGFR ≥ 60 ml/min/1.73m^2^); CKD st.3 (eGFR 30–59 ml/min/1.73m^2^); and CKD st.4–5 (eGFR ≤ 29 ml/min/1.73m^2^) [20].

### Ocular examination

A series of ocular examinations were performed on each patient, including best-corrected visual acuity (BCVA) assessment using Snellen chart, and converted to LogMar for analysis purpose, non-contact tonometry, slit-lamp biomicroscopy, dilated fundus examination, fundus photography, and optical coherence tomography (OCT/OCT-Angiography). Retinal neurovascular parameters were assessed using OCT/OCT-A scans that were conducted by the same expert examiner, and two researchers then evaluated each scan quality independently. The Cirrus HD-OCT 5000 with AngioPlex software (Carl Zeiss Meditec, Dublin, California, USA; V.10.0) was used to acquire macular angiography imaging with 6 × 6 mm scan (perifovea) under pupil dilation. The software automatically provided quantitative measurements according to the Early Treatment DR Society regarding retinal microvascular assessment that, in this study, is represented by vessel density (VD Central, Inner, Outer, Full) and foveal avascular zone (FAZ area and FAZ perimeter) of the superficial capillary plexus. As for the retinal neural parameter, we identified the average of macular ganglion cell-inner plexiform layer thickness (ave mGC-IPLt) and the average of retinal nerve fiber layer thickness (aveRNFLt) that were also obtained automatically. The severity of DR was graded according to The International Clinical Diabetic Retinopathy and Diabetic Macular Edema Disease Severity Scales [21] and classified as No DR, Non Proliverative DR (NPDR), or Proliverative DR (PDR) by a vitreoretinal specialist.

### Measurement of systemic arterial stiffness

Assessment of arterial stiffness was done by measuring brachial-ankle pulse wave velocity (baPWV) and ankle-brachial index (ABI) that were calculated automatically in supine position using Vasera VS-1000 (Fukuda Denshi Co. Ltd, Tokyo, Japan) after at least a 5 min rest period, as done previously by our study group [22]. The baPWV and ABI assessment were recorded from both sides and the average was used for further analysis. In this study, cases were divided into baPWV category [8] (baPWV ≤ 14 and baPWV > 14) and ABI category [23] (abnormal ABI (≤ 0.90 and > 1.40) and normal ABI (0.91–1.40)).

### Statistical analysis

Both eyes were taken into account. Stata version 14 (StataCorp, College Station, TX, USA) was used to perform all the statistical analyses. Categorical data was expressed as an absolute number and percentage, while continuous data as mean ± standard deviation. Regression analysis with multiple hypothesis testing commands with Bonferroni correction, which can perform bootstrap resampling and calculate Westfall-Young step-down adjusted *p*-values, the control of the family-wise error rate, and the allowance for dependence among *p*-values, was used to analyze the comparisons between the groups based on DR severity, renal impairment severity (eGFR level), and arterial stiffness (baPWV and ABI) category. We used Spearman’s correlation test to investigate the associations between arterial stiffness (baPWV and ABI) and retinal neurovascular parameters. In all statistical analyses, statistical significance was assumed if *p* value < 0.05.

## Results

A total of 201 patients were included in this study; however, 31 patients were not willing to undergo evaluation thoroughly. From 170 patients, both eyes (340 eyes) were taken into account. Of these, 75 eyes were of poor-quality image or incomplete data, leaving 265 eyes with complete data and measurable images that were included in the final analysis. The mean age of enrolled patients was 54.43 ± 9.59 years and consisted of 76 (57.14%) females and 57 (42.86%) males. The mean of DM duration was 6.21 ± 6.37 years and the mean of HbA1c was 8.44 ± 2.06%. The mean eGFR and creatinine serum were 66.78 ± 32.80 ml/min/1.73m^2^ and 1.30 ± 1.10 mg/dL, respectively. The mean of arterial stiffness assessed by baPWV and ABI were 15.49 ± 3.07 m/s and 1.05 ± 0.12, respectively. We have summarized the demographic, baseline clinical, laboratory, arterial stiffness, ocular, and OCT/OCTA derived metric characteristics of the patients according to the severity of diabetic retinopathy in Table 1, according to eGFR level in Table 2, according to arterial stiffness (baPWV and ABI) in Tables 3 and 4, respectively, and the correlation of arterial stiffness with retinal neurovascular parameter in Table 5.

**Table 1:** Demographic, laboratory and clinical parameters according to the severity of diabetic retinopathy

	Characteristics	Overall	No DR	NPDR	PDR	*p*-value	Adj.*p*-value
**1**	**Demographic and Baseline**						
	Age (years)	54.43 ± 9.59	54.99 ± 10.55	54.45 ± 8.52	51.83 ± 8.09	0.15	0.44
	Sex male (n,%)	113 (42.64%)	61 (23.02%)	40 (15.09%)	12 (4.53%)	0.44	0.68
	HT (n,%)	109 (41.13%)	43 (16.23%)	48 (18.11%)	18 (6.79%)	0.0007	*0.004
	DM Duration (years)	6.21 ± 6.37	5.88 ± 7.01	6.39 ± 5.50	7.03 ± 6.12	0.33	0.68
	Systolic BP (mmHg)	142.95 ± 21.68	138.8 ± 19.22	144.21 ± 22.07	157.47 ± 24.60	< 0.0001	*0.0002
	Diastolic BP (mmHg)	88.05 ± 11.78	86.09 ± 11.82	88.78 ± 10.08	94.4 ± 14.49	0.0004	*0.003
	Body Mass Index (Kg/m2)	25.46 ± 4.81	26.34 ± 5.15	24.76 ± 4.19	23.87 ± 4.50	0.002	*0.008
	Heart Rate (bpm)	78.18 ± 12.44	76.58 ± 12.51	78.6 ± 12.48	83.93 ± 10.32	0.004	*0.02
**2**	**HbA1C and Lipid Profiles**						
	HbA1C (%)	8.44 ± 2.06	7.99 ± 1.71	8.70 ± 2.16	9.48 ± 2.61	< 0.0001	*0.0003
	Total Cholesterol (mg/dL)	203.11 ± 47.66	201.02 ± 52.30	200.07 ± 43.51	221.65 ± 32.15	0.12	0.47
	HDL-Cholesterol (mg/dL)	54.35 ± 23.75	55.09 ± 23.44	54.11 ± 23.55	51.64 ± 26.33	0.50	0.99
	LDL-Cholesterol (mg/dL)	124.23 ± 39.08	122.78 ± 42.35	121.79 ± 35.47	138.24 ± 31.84	0.16	0.48
	Triglyceride (mg/dL)	160.58 ± 98.88	157.84 ± 99.89	160.16 ± 95.74	173.6 ± 105.88	0.49	0.99
**3**	**Renal Function**						
	eGFR (mL/min/1.73m2)	66.78 ± 32.80	76.38 ± 34.12	58.74 ± 29.49	50.07 ± 22.37	< 0.0001	*<0.0001
	Creatinine (mg/dL)	1.30 ± 1.10	1.03 ± 0.42	1.54 ± 1.44	1.75 ± 1.57	< 0.0001	*<0.0001
**4**	**Arterial Stiffness**						
	Brachial-ankle PWV (m/s)	15.49 ± 3.07	14.77 ± 2.64	15.74 ± 3.11	17.93 ± 3.42	< 0.0001	*<0.0001
	Ankle Brachial Index (ABI)	1.05 ± 0.12	1.04 ± 0.14	1.06 ± 0.12	1.07 ± 0.07	0.34	0.34
**5**	**Ophthalmic Examination**						
	BCVA (LogMAR)	0.19 ± 0 0.43	0.09 ± 0.29	0.17 ± 0.39	0.66 ± 0.69	< 0.0001	*<0.0001
	Spheriqal Equivalent	-0.002 ± 1.72	-0.02 ± 1.77	-0.13 ± 1.67	0.53 ± 1.58	0.45	0.45
	Intraocular Pressure (mmHg)	16.87 ± 2.90	17.10 ± 3.19	16.84 ± 2.53	15.86 ± 2.53	0.05	0.11
**6**	**Retinal Neurovascular**						
	Ave mGC-IPLt (µm)	78.07 ± 14.17	79.49 ± 10.99	77.7 ± 13.28	72.44 ± 25.91	0.02	0.09
	Ave RNFLt (µm)	99.16 ± 18.85	98.25 ± 12.88	95.69 ± 19.46	116.75 ± 30.69	0.005	*0.02
	Vessel Density (VD) (%)						
	VD Central	8.25 ± 3.32	8.33 ± 3.13	8.06 ± 3.39	8.53 ± 3.91	0.96	1.00
	VD Inner	16.83 ± 2.41	17.26 ± 2.34	16.67 ± 2.31	15.45 ± 2.55	0.0001	*0.001
	VD Outer	17.15 ± 2.09	17.49 ± 2.17	16.91 ± 1.97	16.37 ± 1.89	0.002	*0.01
	VD Full	16.83 ± 2.10	17.19 ± 2.13	16.61 ± 2.00	15.95 ± 1.97	0.0009	*0.007
	Fovea Avascular Zone (FAZ)						
	FAZ Area (mm^2^)	0.27 ± 0.13	0.28 ± 0.12	0.27 ± 0.13	0.27 ± 0.16	0.65	1.00
	FAZ Perimeter	2.27 ± 1.26	2.31 ± 1.62	2.20 ± 0.68	2.27 ± 0.84	0.68	1.00
	FAZ Circularity Index	0.68 ± 0.12	0.71 ± 0.10	0.67 ± 0.12	0.62 ± 0.12	< 0.0001	*0.0007

Demographic, laboratory and clinical parameters according to the severity of diabetic retinopathy. Data are expressed as an absolute number or mean ± standard deviation (95% confidence interval). Ave mGC-IPLt, average macular ganglion cell -inner plexiform layer thickness; Ave RNFLt, average retinal nerve fiber layer thickness; BCVA, best corrected visual acuity; BP, blood pressure; DM, diabetes mellitus; eGFR, estimated glomerular filtration rate; FAZ, fovea avascular zone; HT, hypertension; LogMAR, logarithm of the minimum angle of resolution; No DR, no diabetic retinopathy; NPDR, non proliverative diabetic retinopathy; PDR, proliverative diabetic retinopathy; PWV, pulse wave velocity; VD, vessel density

* significant *p*-value

Adj.*p*-value: Adjusted *p*-value (Bonferroni Correction)

**Table 2:** Demographic, laboratory and clinical parameters according to renal impairment category

	Characteristics	Overall	CKD St.1–2	CKD St.3	CKD St.4–5	*p*-value	Adj.*p*-value
**1**	**Demographic and Baseline**						
	Age (years)	54.43 ± 9.59	53.16 ± 10.10	57.14 ± 9.03	52.41 ± 8.41	0.24	0.57
	Sex male (n,%)	113 (42.64%)	52 (20.39%)	45 (17.65%)	12 (4.71%)	0.09	0.34
	HT (n,%)	109 (41.13%)	47 (18.43%)	39 (15.29%)	17 (6.67%)	0.002	*0.01
	DM Duration (years)	6.21 ± 6.37	5.01 ± 5.57	7.31 ± 7.03	9.64 ± 6.72	< 0.0001	*0.0004
	Systolic BP (mmHg)	142.95 ± 21.68	138.77 ± 18.06	144.09 ± 21.73	160.74 ± 29.59	< 0.0001	*<0.0001
	Diastolic BP (mmHg)	88.05 ± 11.78	86.60 ± 10.23	87.29 ± 11.67	95.70 ± 15.48	0.002	*0.01
	Body Mass Index (Kg/m2)	25.46 ± 4.81	25.66 ± 5.14	25.56 ± 4.12	24 ± 5.57	0.19	0.57
	Heart Rate (bpm)	78.18 ± 12.44	78.93 ± 11.14	74.69 ± 12.41	84.85 ± 16.38	0.62	0.62
**2**	**HbA1C and Lipid Profiles**						
	HbA1C (%)	8.44 ± 2.06	8.29 ± 2.04	8.41 ± 1.67	9.19 ± 3.03	0.08	0.33
	Total Cholesterol (mg/dL)	203.11 ± 47.66	202.13 ± 45.12	206.93 ± 53.31	198.42 ± 42.38	0.91	1.00
	HDL-Cholesterol (mg/dL)	54.35 ± 23.75	55.27 ± 22.17	56.23 ± 27.39	42.04 ± 14.79	0.11	0.33
	LDL-Cholesterol (mg/dL)	124.23 ± 39.08	125.85 ± 36.37	122.59 ± 44.06	122.15 ± 38.24	0.53	1.00
	Triglyceride (mg/dL)	160.58 ± 98.88	138.66 ± 75.36	190.84 ± 125.13	187.92 ± 88.09	0.0002	*0.001
**3**	**Renal Function**						
	eGFR (mL/min/1.73m2)	66.78 ± 32.80	88.58 ± 26.74	45.27 ± 8.75	20.70 ± 7.15	< 0.0001	*<0.0001
	Creatinine (mg/dL)	1.30 ± 1.10	0.82 ± 0.22	1.45 ± 0.29	3.39 ± 2.33	< 0.0001	*<0.0001
**4**	**Arterial Stiffness**						
	Brachial-ankle PWV (m/s)	15.49 ± 3.07	14.68 ± 2.75	15.81 ± 2.95	18.12 ± 2.92	< 0.0001	*<0.0001
	Ankle Brachial Index (ABI)	1.05 ± 0.12	1.05 ± 0.12	1.04 ± 0.13	1.08 ± 0.08	0.72	0.72
**5**	**Ophthalmic Examination**						
	BCVA (LogMAR)	0.19 ± 0 0.43	0.15 ± 0.41	0.23 ± 0.47	0.26 ± 0.40	0.12	0.35
	Spheriqal Equivalent	-0.002 ± 1.72	-0.06 ± 1.77	-0.03 ± 1.76	0.41 ± 1.08	0.36	0.73
	Intraocular Pressure (mmHg)	16.87 ± 2.90	16.70 ± 2.62	17.01 ± 2.89	16.98 ± 2.79	0.44	0.73
**6**	**Retinal Neurovascular**						
	Ave mGC-IPL (µm)	78.07 ± 14.17	77.6 ± 13.11	78.91 ± 13.66	75.63 ± 21.41	0.87	1.00
	Ave RNFLt (µm)	99.16 ± 18.85	101.16 ± 18.75	97.67 ± 16.99	90.67 ± 25.29	0.01	0.09
	Vessel Density (VD) (%)						
	VD Central	8.25 ± 3.32	8.28 ± 3.33	7.99 ± 3.21	8.76 ± 3.91	0.83	1.00
	VD Inner	16.83 ± 2.41	17.13 ± 2.05	16.67 ± 2.74	15.52 ± 2.96	0.002	*0.01
	VD Outer	17.15 ± 2.09	17.49 ± 1.75	16.95 ± 2.34	15.72 ± 2.65	< 0.0001	*0.0005
	VD Full	16.83 ± 2.10	17.15 ± 1.75	16.64 ± 2.35	15.49 ± 2.68	0.0002	*0.001
	Fovea Avascular Zone (FAZ)						
	FAZ Area (mm^2^)	0.27 ± 0.13	0.28 ± 0.13	0.28 ± 0.14	0.21 ± 0.10	0.09	0.43
	FAZ Perimeter	2.27 ± 1.26	2.33 ± 1.59	2.26 ± 0.72	1.90 ± 0.58	0.17	0.68
	FAZ Circularity Index	0.68 ± 0.12	0.69 ± 0.11	0.68 ± 0.13	0.69 ± 0.08	0.71	1.00

Demographic, laboratory and clinical parameters according to the severity of renal impairment. Data are expressed as an absolute number or mean ± standard deviation (95% confidence interval). CKD St.1–2 (eGFR ≥ 60 ml/min/1.73m^2^); CKD st.3 (eGFR 30–59 ml/min/1.73m^2^); CKD st.4–5 (eGFR ≤ 29 ml/min/1.73m^2^); Ave mGC-IPLt, average macular ganglion cell -inner plexiform layer thickness; Ave RNFLt, average retinal nerve fiber layer thickness; BCVA, best corrected visual acuity; BP, blood pressure; DM, diabetes mellitus; eGFR, estimated glomerular filtration rate; FAZ, fovea avascular zone; HT, hypertension; LogMAR, logarithm of the minimum angle of resolution; PWV, pulse wave velocity; VD, vessel density

* significant *p*-value

Adj.*p*-value: Adjusted *p*-value (Bonferroni Correction)

**Table 3:** Demographic, laboratory and clinical parameters according to baPWV category

	Characteristics	Overall	baPWV≤14	baPWV > 14	*p*-value	Adj.*p*-value
**1**	**Demographic and Baseline**					
	Age (years)	54.43 ± 9.59	50.68 ± 10.50	56.67 ± 8.2	< 0.0001	*<0.0001
	Sex male (n,%)	113 (42.64%)	42 (15.85%)	71 (26.79%)	0.96	0.96
	HT (n,%)	109 (41.13%)	22 (8.30%)	87 (32.83%)	< 0.0001	*<0.0001
	DM Duration (years)	6.21 ± 6.37	5.55 ± 5.94	6.60 ± 6.59	0.19	0.39
	Systolic BP (mmHg)	142.95 ± 21.68	129.84 ± 14.68	150.77 ± 21.43	< 0.0001	*<0.0001
	Diastolic BP (mmHg)	88.05 ± 11.78	82.69 ± 9.92	91.24 ± 11.67	< 0.0001	*<0.0001
	Body Mass Index (Kg/m2)	25.46 ± 4.81	26.35 ± 4.89	24.94 ± 4.69	0.02	0.06
	Heart Rate (bpm)	78.18 ± 12.44	75.36 ± 13.26	79.85 ± 11.64	0.004	*0.02
**2**	**HbA1C and Lipid Profiles**					
	HbA1C (%)	8.44 ± 2.06	8.36 ± 1.94	8.48 ± 2.13	0.67	1.00
	Total Cholesterol (mg/dL)	203.11 ± 47.66	203.78 ± 49.95	202.68 ± 46.30	0.86	1.00
	HDL-Cholesterol (mg/dL)	54.35 ± 23.75	54.22 ± 22.64	54.43 ± 24.52	0.94	1.00
	LDL-Cholesterol (mg/dL)	124.23 ± 39.08	128.83 ± 39.57	121.34 ± 38.62	0.14	0.56
	Triglyceride (mg/dL)	160.58 ± 98.88	141.43 ± 68.02	172.88 ± 112.91	0.01	0.07
**3**	**Renal Function**					
	eGFR (mL/min/1.73m2)	66.78 ± 32.80	81.57 ± 36.96	58.01 ± 26.51	< 0.0001	*<0.0001
	Creatinine (mg/dL)	1.30 ± 1.10	0.99 ± 0.42	1.49 ± 1.32	0.0004	*0.0004
**4**	**Arterial Stiffness**					
	Ankle Brachial Index (ABI)	1.05 ± 0.12	1.04 ± 0.13	1.06 ± 0.12	0.28	0.28
**5**	**Ophthalmic Examination**					
	BCVA (LogMAR)	0.19 ± 0 0.43	0.10 ± 0.31	0.24 ± 0.48	0.009	*0.03
	Spheriqal Equivalent	-0.002 ± 1.72	-0.19 ± 1.55	0.13 ± 1.82	0.24	0.47
	Intraocular Pressure (mmHg)	16.87 ± 2.90	16.9 ± 2.79	16.84 ± 2.98	0.85	0.85
**6**	**Retinal Neurovascular**					
	Ave mGC-IPL (µm)	78.07 ± 14.17	79.77 ± 11.84	77.02 ± 15.37	0.12	0.48
	Ave RNFLt (µm)	99.16 ± 18.85	97.06 ± 14.10	100.45 ± 21.20	0.17	0.49
	Vessel Density (VD) (%)					
	VD Central	8.25 ± 3.32	8.92 ± 3.22	7.85 ± 3.32	0.01	0.06
	VD Inner	16.83 ± 2.41	17.82 ± 1.51	16.25 ± 2.65	< 0.0001	*<0.0001
	VD Outer	17.15 ± 2.09	18.02 ± 1.47	16.62 ± 2.24	< 0.0001	*<0.0001
	VD Full	16.83 ± 2.10	17.74 ± 1.42	16.29 ± 2.26	< 0.0001	*<0.0001
	Fovea Avascular Zone (FAZ)					
	FAZ Area (mm^2^)	0.27 ± 0.13	0.28 ± 0.11	0.26 ± 0.14	0.25	0.49
	FAZ Perimeter	2.27 ± 1.26	2.41 ± 1.83	2.18 ± 0.70	0.18	0.49
	FAZ Circularity Index	0.68 ± 0.12	0.71 ± 0.11	0.67 ± 0.12	0.03	0.13

Demographic, laboratory and clinical parameters according to baPWV category. Data are expressed as an absolute number or mean ± standard deviation (95% confidence interval). Ave mGC-IPLt, average macular ganglion cell -inner plexiform layer thickness; Ave RNFLt, average retinal nerve fiber layer thickness; baPWV, brachial-ankle pulse wave velocity; BCVA, best corrected visual acuity; BP, blood pressure; DM, diabetes mellitus; eGFR, estimated glomerular filtration rate; FAZ, fovea avascular zone; HT, hypertension; LogMAR, logarithm of the minimum angle of resolution; PWV, pulse wave velocity; VD, vessel density

* significant *p*-value

Adj.*p*-value: Adjusted *p*-value (Bonferroni Correction)

**Table 4:** Demographic, laboratory and clinical parameters according to ABI category

	Characteristics	Overall	Abnormal ABI	Normal ABI	*p*-value	Adj.*p*-value
**1**	**Demographic and Baseline**					
	Age (years)	54.43 ± 9.59	52.62 ± 7.42	54.65 ± 9.81	0.28	0.73
	Sex male (n,%)	113 (42.64%)	6 (2.26%)	107 (40.38%)	0.01	0.07
	HT (n,%)	109 (41.13%)	9 (3.40%)	100 (37.74%)	0.24	0.73
	DM Duration (years)	6.21 ± 6.37	7.14 ± 6.07	6.09 ± 6.41	0.41	0.73
	Systolic BP (mmHg)	142.95 ± 21.68	149.41 ± 32.35	142.16 ± 19.94	0.09	0.44
	Diastolic BP (mmHg)	88.05 ± 11.78	83.21 ± 15.46	88.64 ± 11.14	0.02	0.11
	Body Mass Index (Kg/m2)	25.46 ± 4.81	29.52 ± 7.13	24.97 ± 4.20	< 0.0001	*<0.0001
	Heart Rate (bpm)	78.18 ± 12.44	81.38 ± 13.95	77.78 ± 12.21	0.14	0.57
**2**	**HbA1C and Lipid Profiles**					
	HbA1C (%)	8.44 ± 2.06	7.81 ± 1.76	8.52 ± 2.08	0.08	0.24
	Total Cholesterol (mg/dL)	203.11 ± 47.66	237.86 ± 67.47	198.49 ± 42.48	< 0.0001	*0.0001
	HDL-Cholesterol (mg/dL)	54.35 ± 23.75	57.45 ± 29.59	53.93 ± 22.89	0.46	0.91
	LDL-Cholesterol (mg/dL)	124.23 ± 39.08	150.27 ± 45.55	120.79 ± 36.91	0.0001	*0.0003
	Triglyceride (mg/dL)	160.58 ± 98.88	161.62 ± 84.85	160.44 ± 100.76	0.95	0.95
**3**	**Renal Function**					
	eGFR (mL/min/1.73m2)	66.78 ± 32.80	65.48 ± 30.89	66.95 ± 33.10	0.82	1.00
	Creatinine (mg/dL)	1.30 ± 1.10	1.19 ± 0.71	1.32 ± 1.14	0.56	1.00
**4**	**Arterial Stiffness**					
	Brachial-ankle PWV (m/s)	15.49 ± 3.07	15 ± 4.10	15.56 ± 2.92	0.35	0.35
**5**	**Ophthalmic Examination**					
	BCVA (LogMAR)	0.19 ± 0.43	0.19 ± 0.41	0.19 ± 0.43	0.99	0.99
	Spheriqal Equivalent	-0.002 ± 1.72	-0.49 ± 2.06	0.06 ± 1.66	0.17	0.33
	Intraocular Pressure (mmHg)	16.87 ± 2.90	17.70 ± 3.47	16.77 ± 2.82	0.10	0.31
**6**	**Retinal Neurovascular**					
	Ave mGC-IPL (µm)	78.07 ± 14.17	79.48 ± 8.63	77.89 ± 14.72	0.57	1.00
	Ave RNFLt (µm)	99.16 ± 18.85	97.89 ± 15.59	99.33 ± 19.28	0.70	1.00
	Vessel Density (VD) (%)					
	VD Central	8.25 ± 3.32	6.74 ± 3.29	8.43 ± 3.28	0.009	0.08
	VD Inner	16.83 ± 2.41	16.37 ± 2.61	16.89 ± 2.38	0.27	1.00
	VD Outer	17.15 ± 2.09	17.11 ± 2.49	17.15 ± 2.05	0.92	1.00
	VD Full	16.83 ± 2.10	16.67 ± 2.46	16.85 ± 2.06	0.67	1.00
	Fovea Avascular Zone (FAZ)					
	FAZ Area (mm^2^)	0.27 ± 0.13	0.32 ± 0.15	0.27 ± 0.13	0.02	0.18
	FAZ Perimeter	2.27 ± 1.26	2.38 ± 0.75	2.25 ± 1.31	0.61	1.00
	FAZ Circularity Index	0.68 ± 0.12	0.70 ± 0.12	0.68 ± 0.12	0.54	1.00

Demographic, laboratory and clinical parameters according to ABI category. Data are expressed as an absolute number or mean ± standard deviation (95% confidence interval). ABI, ankle-brachial index; Abnormal ABI (ABI ≤ 0.90 and > 1.40); Normal ABI (ABI 0.91–1.40); Ave mGC-IPLt, average macular ganglion cell -inner plexiform layer thickness; Ave RNFLt, average retinal nerve fiber layer thickness; baPWV, brachial-ankle pulse wave velocity; BCVA, best corrected visual acuity; BP, blood pressure; DM, diabetes mellitus; eGFR, estimated glomerular filtration rate; FAZ, fovea avascular zone; HT, hypertension; LogMAR, logarithm of the minimum angle of resolution; PWV, pulse wave velocity; VD, vessel density

* significant *p*-value

Adj.*p*-value: Adjusted *p*-value (Bonferroni Correction)

**Table 5:** Correlation between arterial stiffness and retinal neurovascular parameter

Retinal Neurovascular Parameter	*p*-value baPWV	r-baPWV	*p*-value ABI	r-ABI
Ave mGC-IPLt	*0.0034	-0.19	0.78	-0.02
Ave RNFLt	0.59	0.03	0.68	0.03
Vessel Density (VD)				
VD Central	0.14	-0.09	***<0.0001	0.27
VD Inner	***<0.0001	-0.29	*0.008	0.17
VD Outer	***<0.0001	-0.34	0.93	0.006
VD Full	***<0.0001	-0.34	0.43	0.053
Fovea Avascular Zone (FAZ)				
FAZ Area	0.10	-0.11	**0.0005	-0.23
FAZ Perimeter	0.60	-0.03	*0.002	-0.21
FAZ Circularity Index	*0.009	-0.17	0.99	0.0006

ABI, ankle-brachial index; Ave mGC-IPLt, average macular ganglion cell-inner plexiform layer thickness; Ave RNFLt, average retinal nerve fiber layer thickness; baPWV, brachial-ankle pulse wave velocity; *p < 0.05 **p < 0.001 ***p < 0.0001

### The comparisons among the groups according to the severity of DR

The comparisons according to the severity of DR shown in Table 1 were classified as No DR (135; 50.94%), NPDR (100; 37,74%), and PDR (30; 11.32%). We found that HT, SBP, DBP, BMI, HR, HbA1c, eGFR, and Cr showed significant difference between each group. In the more severe group (PDR), eGFR was more likely to decrease significantly. The mean of eGFR was 76.38 ± 34.12 in No DR, 58.74 ± 29.49 in NPDR, and 50.07 ± 22.37 in the PDR group (*p* < 0.0001). In terms of arterial stiffness, only baPWV showed significant difference while ABI was comparable among three groups. The mean of baPWV was 14.77 ± 2.64 in No DR, 15.74 ± 3.11 in NPDR, and 17.93 ± 3.42 in the PDR group (*p* < 0.0001).

With regard to the ocular and retinal neurovascular parameter, BCVA, aveRNFLt, perifoveal VD (Inner, Outer, Full), and FAZ circularity were significantly different between each group. Among DR groups, the PDR group demonstrated the highest mean of SBP, DBP, HR, HbA1c, total cholesterol, Cr, baPWV, and BCVA as well as the lowest mean of BMI, eGFR, VD (Inner, Outer, Full), and FAZ circularity.

### The comparisons among the groups according to the severity of renal impairment (eGFR level)

Table 2 demonstrates the comparisons of all parameters according to eGFR. Of the 265 eyes, 55.69% were classified as CKD st.1–2, 33.72% as CKD st.3 and 10.59% as CKD st.5. The HT, DM duration, SBP, DBP, HR, TG, Cr, and BCVA differed significantly between each CKD group. Patients with more severe CKD were more likely to have longer DM duration (*p* < 0.001), higher levels of SBP (*p* < 0.0001), DBP (*p* = 0.01) and Cr (*p* < 0.0001). However, there were no significant differences with regard to BMI, HbA1c, total cholesterol, HDL, LDL, ABI, BCVA, SE, and IOP among the three groups.

In terms of arterial stiffness, baPWV was found to be significantly higher in CKD st.4–5 group; nonetheless, ABI was comparable among different groups. The mean of baPWV was 14.68 ± 2.75 in CKD st.1–2, 15.81 ± 2.95 in CKD st.3, and 18.12 ± 2.92 in CKD st.4–5 group (*p* < 0.0001). Regarding the retinal neurovascular parameters, only perifoveal VD (Inner, Outer, Full) showed significant differences that were more likely to decrease following the decrease of eGFR. However, ave mGC-IPLt and aveRNFLt, that tend to decrease in more severe CKD, VD central, and FAZ were not significantly different between the groups.

### The comparisons among the groups according to arterial stiffness (baPWV and ABI) category

Arterial stiffness category was classified according to baPWV category (Table 3) and ABI category (Table 4). Of the 265 eyes, 99 (37.36%) were classified as baPWV≤ 14 and 166 (62.64%) as baPWV > 14. The age, HT, SBP, DBP, and HR were significantly higher while BMI was nearly significantly lower in baPWV > 14 group. The mean of eGFR was found to be significantly lower in baPWV > 14 group (81.57 ± 36.96 and 58.01 ± 26.51, for baPWV≤14 and baPWV > 14, respectively, *p* < 0.0001). Moreover, retinal neurovascular parameter comparison showed that VD (Inner, Outer, Full) were significantly lower in baPWV > 14 group (*p* < 0.0001). On the other hand, ave mGC-IPLt and FAZ, despite their tendency to decrease exhibit no significant differences between the two baPWV group.

According to ABI category (Table 4), of the 265 eyes, 29 (10.94%) were classified as Abnormal ABI in which abnormally low or high ABI (ABI≤ 0.90 or > 1.40) and 236 (89.06%) were classified as Normal ABI (0.91–1.40). The mean eGFR was comparable between the two ABI groups. In terms of retinal neurovascular parameter, we noted decreased aveRNFLt, VD and wider FAZ in the abnormal ABI group, however there were no significant differences.

### Correlation between arterial stiffness and retinal neurovascular parameters

The results of Spearman’s correlation analysis (Table 5) demonstrated that baPWV was significantly negatively correlated with ave mGC-IPL, VD (Inner, Outer, Full), and FAZ circularity. While ABI showed a significant positive correlation with VD (Central, Inner), it negatively correlated with FAZ (FAZ area and perimeter).

## Discussion

In the current study, we investigated the association between retinal neurovascular parameter, derived from quantitative OCT/OCTA metrics, and systemic arterial stiffness, as measured by baPWV and ABI, in type 2 DM patients with varying degrees of renal impairment from a single center in Indonesia. Patients with more severe renal impairment were more likely to have longer DM duration, higher baPWV value, and decreased perifovea VD. This is in accordance with the findings in the more severe DR group, in which eGFR was more likely to decrease significantly with higher baPWV value along with the changes in VD and FAZ. This is similar to what we found in the baPWV > 14 group, where the mean of eGFR and VD were significantly lower while renal impairment was found comparable between the two ABI group. Moreover, baPWV was significantly negatively correlated with ave mGC-IPLt, VD, and FAZ, while ABI showed a significant positive correlation with VD; on the other hand, it negatively correlated with FAZ. Our findings confirmed the close association between renal impairment, diabetic retinopathy, arterial stiffness, and retinal neurovascular parameter, thus supporting the potential of OCT/OCTA derived parameters and baPWV measurement to detect early microvascular changes in the retina that might mimic the microvascular alteration in kidney and systemic arterial stiffness in type 2 DM.

There have been several publications referencing studies on systemic arterial stiffness in type 2 DM patients. One recent study by Xueyang Zhang et al. found substantially greater baPWV in the group with early diabetic nephropathy (DN) in comparison to diabetics without nephropathy group, and suggested baPWV as a repeatable technique for diagnosing early DN [13]. Its conclusion is consistent with our findings that greater baPWV (baPWV > 14) groups had lower eGFR values than other groups, indicating decreased renal function. Other studies were focusing on the association between arterial stiffness and the presence or the severity of DR [14, 15] in association with arterial stiffness using different methods of assessment as well as different parameter such as cfPWV [16], ABI [14], and CAVI [17]. Meanwhile, we discovered that the most severe type of DR had the greatest baPWV and lowest eGFR values, as well as retinal microvascular changes evidenced by declining VD and FAZ circularity. Our findings were consistent with a prospective study by Yaxin An et al., in which participants had new onset or progression of DR after a mean follow-up of 16.4 months; furthermore, baseline baPWV may be an independent predictor of DR progression, suggesting that increased arterial stiffness may contribute to the development of DR [15]. Additionally, one study demonstrated the relationship between abnormal ABI and PDR, which also showed declining renal function [14]. Therefore, eGFR results can be used by doctors to monitor patients and enable fast referrals to ophthalmologists for quick care of sight-threatening DR [24].

The association between renal function or early CKD and retinal neural or microvascular changes derived from OCT/OCTA in type 2 DM were the subject of some prior investigations [10–12]. In our study, we found that patients with more severe CKD were more likely to have longer DM duration, higher baPWV, as well as decreasing VD. This finding was in accordance with the cases in PDR group which demonstrated the highest baPWV, lowest eGFR value, retinal vascular alteration evidenced by decreasing VD and FAZ circularity, as well as decrease VD according to baPWV group. Our findings support the results from Oliveira da Silva et al., which suggested early neurovascular damage in patients with type 2 DM and the changes were more significant in patient with DKD [12]. Additionally, Kim et al., explained the relationship between chorioretina microvasculature and arterial stiffness assessed by CAVI, and showed that there was a substantial link between arteriosclerosis and choroidal vascular abnormalities in DR [17]. Our results support the concept that DM, CKD, and arterial stiffness may have impact on the retinal neurovascular system. The varied study populations, study designs that involved patients with various DM durations and severity levels, as well as clinical characteristics, may have contributed to the inconsistent results or disparity.

Patients with chronic renal disease, metabolic syndrome, hypertension, diabetes, sleep apnoea syndrome, aging, tachycardia, and post-menopausal symptoms have all been linked to an increase in baPWV. Additionally, it has been discovered that a greater baPWV is linked to more advanced organ damage in those with diabetes and hypertension, and that a 1 m/s rise in baPWV is linked to a 12% increase in the risk of cardiovascular events [25]. According to Yamashina, a baPWV > 14.0 m/s is an independent variable for the risk stratification based on the Framingham score and for the identification of individuals with atherosclerotic cardiovascular disease [8]. Further, previous reports derived from East Asian countries regarding baPWV measurement demonstrated that it has good reproducibility, is non-invasive in nature, is more convenient, and is also easier compared to cfPWV; further, since the methodology’s generalizability is proven, baPWV is more commonly used and is suitable for screening huge populations [25]. Consequently, baPWV has the potential to be used globally as a marker of cardiovascular risk by measuring arterial stiffness [8, 15, 25].

Microcirculation may play an important role in cardiovascular disease associated with chronic kidney disease. Increasing evidence suggests an association between systemic microvascular dysfunction and unfavorable cardiovascular outcomes [26]. Furthermore, the concept of DR as a microvascular abnormality has changed, placing more emphasis on the involvement of the neurovascular unit and including microvascular changes and the deterioration of neural components in which glial, neural, and microvascular dysfunction are interdependent and are crucial for the development of DR [18, 19]. In this study, we noted that retina neurovascular alteration were associated with a decreased eGFR and high baPWV. This supports the concept that retinal “neurovascular unit” may be affected in DM patients along with the deterioration of renal function and the increase of arterial stiffness. Diabetes causes microvascular damage that predominantly affects the ganglion cells and their axons, which results in reduced capillary density or FAZ disruption [12, 17]. Further, damage to either the neuronal or vascular components of the retinal neurovascular unit will result in the loss of autoregulation in the retina [18]. Several potential causes for the neural damage in CKD patients could be related to DM, hypertension, increasing neuroinflammation, oxidative stress, vascular dysfunction, or neuron and astrocyte death [27].

In individuals with type 2 DM, central arterial stiffness was found to be linked to the occurrence and severity of DR, indicating a potential causative role [16]. Pulse wave velocity (PWV > 19.6 m/s) was substantially correlated with the likelihood of PDR and rising PWV was favorably associated with the severity of DR [28]. Furthermore, increased arterial stiffness may also play a role in the development of DR, since baseline baPWV may be an independent predictor of DR progression [15]. Numerous theories have been put forth, but the underlying processes linking central arterial stiffness and the ocular microvasculature, especially DR, are not yet well understood [16, 17]. The development of DR may result from an increase in central artery stiffness that causes a lack of buffering function and a broader forward pulsatile pressure wave to be transmitted. This might disrupt the retinal and choroidal microcirculation [29, 30]. There is also a chance that the common underlying pathologic processes of diabetic mellitus, such as chronic inflammation and endothelial dysfunction, may be the cause of both alterations in the ocular microvasculature and systemic arterial stiffness [31].

### Strengths and limitations

The strength of the current study is that we confirmed the significant association between retinal neurovascular alteration and both macrovascular (arterial stiffness) and microvascular (declining renal function, DR severity) in type 2 DM simultaneously. Our findings imply that the ocular neurovascular unit is prone to early alterations as a result of chronic hyperglycemia, which may mimic kidney microvascular changes and stiffness of major arteries. We encourage the application of OCT/OCTA-derived metrics and baPWV measurement in diabetic patients to evaluate and to monitor the progression of DR, DKD as well as CVD risk factor. Another feature of this study is the homogeneous population from a single center, which represents a real-world setting. Patients attending diabetes and endocrine OPD may have diabetic retinopathy, diabetic kidney disease, or cardiovascular risk factor who might need non-invasive measurement for early diagnosis. As the retina was considered to be a window to the kidney [9], neuroretina and microvascular assessment derived from the OCT/OCTA imaging may play an important role for estimating renal function in diabetic patients [32]. Therefore, combining the non-invasive, convenient, and reproducible measurement of retinal neurovascular (OCT/OCTA) and arterial stiffness (baPWV) as an adjunct to renal function assessment in diabetic patients may be of benefit to support an integrated screening. Findings from these studies will be crucial, not only for increasing our understanding of the pathophysiological connections between the development and progression of macro- and microangiopathy in diabetes, but also to assist in the implementation of a prompt, effective, and personalized strategy for managing diabetes complications, as well as to lower morbidity and mortality.

Several limitations should be taken into account when considering the results of this study. First, due to its cross-sectional nature, the causal relationship between retina neurovascular alteration and either kidney and arterial stiffness cannot be determined. Second, the small sample size for each stage and several potential confounding factors were also unadjusted. Third, some information on albuminuria as another important indicator of renal function and spherical equivalent were missing in most of the patients, and we did not measure the axial length as one of the confounding factors for retinal vessel density and FAZ assessment. Fourth, systemic arteriosclerosis and DR may have similar risk factors, and both conditions may result from the same pathogenic mechanism in DM. As a result, the correlations between baPWV and ocular microvascular alterations may be underestimated. Nonetheless, our findings can serve as the foundation for further study. There is still a long way for us to go in order to explore the relationship between retinal neurovascular changes, kidneys, and systemic arterial stiffness. Future studies should address all those limitations. A larger number of diabetic patients with a wide range of renal impairment should be investigated in a prospective longitudinal study to evaluate whether OCTA-derived metrics can predict the progression of chronic kidney disease in diabetes and arterial stiffness as CVD risk factor. Sub-analysis on the association between each retinal neural and microvascular parameter should be performed.

## Conclusions

In summary, our findings confirmed the close association between changes in the retinal neurovascular system, the severity of DR, renal impairment, and arterial stiffness. Our findings may have implications on the routine screening and comprehensive care of type 2 DM patients. In addition, patients with type 2 DM, particularly those with DR and renal function deterioration, should be alert for sub-clinical macroangiopathy. We advise rapid ocular screening, renal function assessment, and arterial stiffness measurement in DM patients. Future longitudinal research is necessary to determine renal function, DR severity, and baPWV measurement, along with retinal neurovascular assessment, which may enhance cardiovascular risk stratification over existing markers.

## Data Availability

The datasets used and/ or analysed during the current study are available from the corresponding author on reasonable request.

## Abbreviations

ABI: Ankle brachial index
aveRNFLt: Average of retinal nerve fiber layer thickness
ave mGC-IPLt: Average macular ganglion cell- inner plexiform layer thickness
baPWV: Brachial-ankle pulse wave velocity
BCVA: Best-corrected visual acuity
BMI: Body mass index
BP: Blood Pressure
CAVI: Cardio-ankle vascular index
cfPWV: Carotid-femoral pulse wave velocity
CKD: Chronic kidney disease
Cr: Creatinine serum
CVD: Cardiovascular disease
DBP: Diastolic blood pressure
DiORS Study: Diabetic Ocular Renal Surabaya Study
DKD: Diabetic kidney disease
DM: Diabetes mellitus
DN: Diabetic nephropathy
DR: Diabetic retinopathy
eGFR: Estimated glomerular filtration rate
FAZ: Foveal avascular zone
HbA1c: Hemoglobin A1c
HDL: High-density lipoprotein
HR: Heart rate
HT: Hypertension
LDL: Low-density lipoprotein
LogMAR: Logarithm of the minimum angle of resolution
MDRD: Modification of diet in renal disease
NPDR: Non Proliverative DR
OCT: Optical coherence tomography
OCT-A: Optical coherence tomography angiography
PDR: Proliverative DR
PWV: Pulse wave velocity
SBP: Systolic blood pressure
TG: Triglycerides
VD: Vessel density
